# Effect of Gray Value Discretization and Image Filtration on Texture Features of the Pancreas Derived from Magnetic Resonance Imaging at 3T

**DOI:** 10.3390/jimaging8080220

**Published:** 2022-08-18

**Authors:** Bassam M. Abunahel, Beau Pontre, Maxim S. Petrov

**Affiliations:** 1 School of Medicine, University of Auckland, Auckland 1023, New Zealand; 2 School of Medical Sciences, University of Auckland, Auckland 1023, New Zealand

**Keywords:** magnetic resonance imaging, image pre-processing, radiomics, pancreas, chronic pancreatitis

## Abstract

Radiomics of pancreas magnetic resonance (MR) images is positioned well to play an important role in the management of diseases characterized by diffuse involvement of the pancreas. The effect of image pre-processing configurations on these images has been sparsely investigated. Fifteen individuals with definite chronic pancreatitis (an exemplar diffuse disease of the pancreas) and 15 healthy individuals were included in this age- and sex-matched case-control study. MR images of the pancreas were acquired using a single 3T scanner. A total of 93 first-order and second-order texture features of the pancreas were compared between the study groups, by subjecting MR images of the pancreas to 7 image pre-processing configurations related to gray level discretization and image filtration. The studied parameters of intensity discretization did not vary in terms of their effect on the number of significant first-order texture features. The number of statistically significant first-order texture features varied after filtering (7 with the use of logarithm filter and 3 with the use of Laplacian of Gaussian filter with 5 mm σ). Intensity discretization generally affected the number of significant second-order texture features more markedly than filtering. The use of fixed bin number of 16 yielded 42 significant second-order texture features, fixed bin number of 128–38 features, fixed bin width of 6–24 features, and fixed bin width of 42–26 features. The specific parameters of filtration and intensity discretization had differing effects on radiomics signature of the pancreas. Relative discretization with fixed bin number of 16 and use of logarithm filter hold promise as pre-processing configurations of choice in future radiomics studies in diffuse diseases of the pancreas.

## 1. Introduction

Diseases of the pancreas—both focal pancreatic lesions (such as pancreatic cancer) and diffuse diseases of the pancreas (such as chronic pancreatitis, diabetes mellitus)—are complex and influenced by numerous environmental, metabolic, and genetic factors [1,2,3,4]. The complex pathogenesis of these diseases makes its investigation quite challenging. The conventional method for radiological investigation of diseases of the pancreas is based on subjective (i.e., qualitative) assessment of medical images obtained using different modalities, one of the most advanced of which is magnetic resonance (MR) imaging [5,6]. There is a growing appreciation that MR images also contain valuable numerical information that could be extracted in an objective (i.e., quantitative) fashion [7]. For example, quantification of pancreatic parenchyma with the use of diffusion-weighted imaging or T1 mapping has been investigated reasonably well [8]. An emerging complementary approach is radiomics, in which target organs are characterized using quantitative parameters such as shape, first-order and second-order texture features [9,10,11]. The application of radiomics to the pancreas is gaining momentum as a comprehensive 2021 systematic review has identified 72 published studies that used radiomics as differential diagnosis, classification, and prediction tools for pre-malignant pancreatic lesions, pancreatic cancers, acute pancreatitis, and type 2 diabetes mellitus [12].

Although promising, radiomics of the pancreas is in its infancy and numerous questions warrant purposely-designed investigations before a robust radiomics signature is developed. One important consideration relates to the use of image pre-processing, which generally aims to enhance image quality before extracting radiomics features. Intensity discretization and image filtration are exemplar types of image pre-processing. The former may particularly be useful when applied to MR images as pixel intensity in these images is often different, with great variability between images [13]. Extraction of texture features from the native gray values is bedeviled by non-informative features. Grouping the native image intensities into a finite number of levels with fewer distinct gray values can reduce the image noise [14]. Absolute discretization using fixed bin size and relative discretization using fixed bin number are the two common methods of intensity discretization [15]. Absolute discretization has been advocated in some studies whereas other studies have found that relative discretization results in more reproducible and robust texture features [16,17,18]. It is worth noting that those studies focused on organs other than the pancreas, and the optimal approach to discretization of pancreas MR images is yet to be determined. Image filtration is another common type of pre-processing. In comparison with other imaging modalities, MR images have both Gaussian and Rician noise and, therefore, may benefit from denoising [19]. Several types of image filters (e.g., Laplacian of Gaussian, logarithm) were applied in earlier radiomics studies of the pancreas [20,21,22,23,24,25]. However, there is a paucity of studies on the effect of specific parameters of filters on radiomics of the pancreas derived from MR images.

The aim of the present study was to investigate the effect of gray level discretization and image filtration on radiomics of the pancreas in two polar states of the pancreas–chronic pancreatitis (an exemplar diffuse disease of the pancreas) and healthy pancreas.

## 2. Methods

### 2.1. Study Population

The study had case-control design and was part of the TUCANA (TextUre of the panCreAs oN imAges) project [26]. Cases were adults with definite chronic pancreatitis drawn from a longitudinal cohort study of individuals after pancreatitis (case group). The diagnosis of chronic pancreatitis required the presence of Cambridge grade ≥ 3 or parenchymal calcifications on modern cross-sectional imaging. The cases did not undergo any intervention involving the pancreas and had no pancreatic duct stones or cysts. The exclusion criteria were detailed elsewhere [27]. The control group included adult healthy volunteers who were matched on age and sex with the case group.

### 2.2. Image Acquisition and Reconstruction

**Figure 1:**
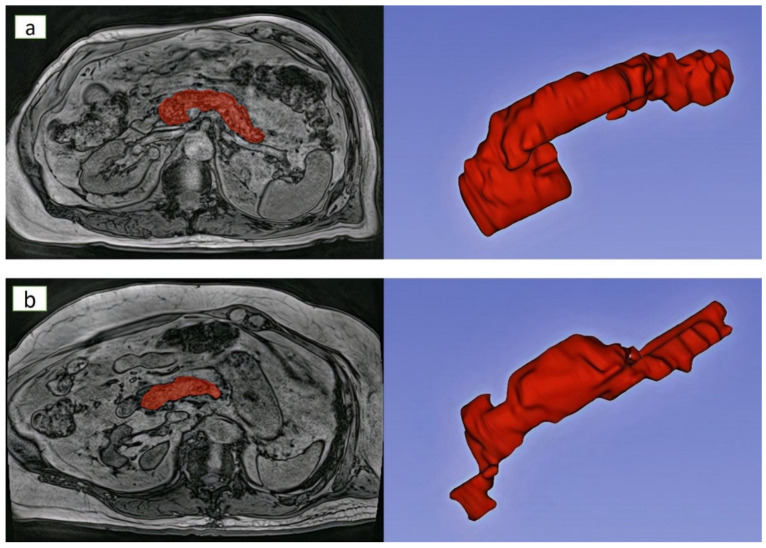
Exemplar segmentation and 3D reconstruction of the pancreas MR images in individuals with (**a**) healthy pancreas and (**b**) chronic pancreatitis. *Footnotes:* The healthy individual was a 77-year-old man with a BMI of 27. The individual with chronic pancreatitis was a 77-year-old man with a BMI of 26.

A 3.0 Tesla MAGNETOM Skyra scanner (Siemens, Erlangen, Germany) was used in all study participants. Abdominal MR scan was carried out with the participant in headfirst supine position. Axial T1-weighted MR images were acquired using a volume-interpolated breath hold examination (VIBE) sequence with the following parameters: echo time, 3.69 ms; repetition time, 3.85 ms; field of view, 420 mm; basic resolution, 320; flip angle, 9°; pixel bandwidth, 920 Hz; voxel size: 0.9 mm × 0.9 mm × 5.0 mm. Only out-of-phase images (owing to their higher contrast) were exported as Digital Imaging and Communications in Medicine files and used for radiomics analysis [28]. The pancreas was manually segmented on every axial slice in which it was visible. The border of the pancreas was carefully delineated to exclude the surrounding abdominal structures from the segmentation (Figure 1).

### 2.3. Image Pre-Processing Configurations

Two methods of intensity discretization were used—absolute discretization and relative discretization. The former was based on applying fixed bin size and the latter—fixed number of bins. A previous study demonstrated that reproducible texture features were extracted with number of bins in the range of 16–128 bins [29]. That is why in the present study the two polar fixed number of bins were used—16 and 128. For absolute discretization, the bin width was fixed at either 6 or 42. Since the range of gray values in our dataset was 0–682, these two bin widths cover range of 16–128 bins. Two image filters were applied-Laplacian of Gaussian filter and logarithm filter. The Gaussian filter was used to smoothen the image and then convolved by the Laplacian filter for edge enhancement. The width of the Gaussian filter is determined by sigma (σ), with small σ emphasising finer texture features and large σ emphasising coarser texture features. In the present study, σ values of 2 mm (fine texture features) and 5 mm (coarse texture features) were investigated.

### 2.4. Radiomics Feature Extraction

3D mask in PyRadiomics (version 3.0, GitHub, San Francisco, CA, USA) was used for extracting radiomics features from all slices containing the pancreas. A total of 18 first-order and 75 second-order texture features were extracted from the pancreas MR images of all study participants [26]. The first-order texture features describe the distribution of image intensities within the pancreas. The second-order texture features, which represent the spatial relationship between the image intensities within the pancreas, were extracted from 5 different image matrices. These included 24 features from Gray Level Co-occurrence Matrix (GLCM), 16 features from Gray Level Run Length Matrix (GLRLM), 16 features from Gray Level Size Zone Matrix (GLSZM), 14 features from Gray Level Dependence Matrix (GLDM), and 5 features from Neighboring Gray Tone Difference Matrix (NGTDM).

### 2.5. Statistical Analysis

All analyses were performed using SPSS (version 27, New York, NY, USA). The differences in the mean values of texture features between the two groups were compared using the independent sample t-test. False discovery rate (an adjusted *p* value) was used to account for multiple comparisons [30,31]. Texture features that had an adjusted *p* value (q value) of less than 0.05 were deemed to be statistically significant.

## 3. Results

### 3.1. Characteristics of Individuals

The case group included 15 individuals with definite chronic pancreatitis, enrolled in a median of 40 months (interquartile range, 11–82 months) after their first attack of pancreatitis. The control group included 15 age- and sex-matched healthy individuals. The two groups had similar characteristics (Table 1).

### 3.2. Features Extracted from Intensity Discretized MR Images of the Pancreas

#### 3.2.1. Intensity Discretization Based on Fixed Bin Numbers of 16 and 128

The discretization of MR image intensities using fixed bin number of 16 resulted in a total of 45 features that showed a statistically significant difference between the two groups (Table 2). These included 3 first-order (Supplementary Table S1) and 42 second-order texture features. Out of the latter, 15 were from GLCM (Supplementary Table S2), 11 were from GLRLM (Supplementary Table S3), 6 were from GLSZM (Supplementary Table S4), 9 were from GLDM (Supplementary Table S5), and 1 was from NGTDM (Supplementary Table S6).

The discretization of MR image intensities using fixed bin number of 128 resulted in a total of 41 features that showed a statistically significant difference between the groups (Table 2). These included 3 first-order (Supplementary Table S1) and 38 second-order texture features. Out of the latter, 12 were from GLCM (Supplementary Table S2), 8 were from GLRLM (Supplementary Table S3), 9 were from GLSZM (Supplementary Table S4), 8 were from GLDM (Supplementary Table S5), and 1 was from NGTDM (Supplementary Table S6).

#### 3.2.2. Intensity Discretization Based on Fixed Bin Widths of 6 and 42

The discretization of MR image intensities using fixed bin width of 6 resulted in a total of 27 features that showed a statistically significant difference between the groups (Table 2). These included 3 first-order (Supplementary Table S1) and 24 second-order texture features. Out of the latter, 4 were from GLCM (Supplementary Table S2), 6 were from GLRLM (Supplementary Table S3), 6 were from GLSZM (Supplementary Table S4), and 8 were from GLDM (Supplementary Table S5).

The discretization of MR image intensities using fixed bin width of 42 resulted in a total of 29 features that showed a statistically significant difference between the groups (Table 2). These included 3 first-order (Supplementary Table S1) and 26 second-order texture features. Out of the latter, 6 were from GLCM (Supplementary Table S2), 8 were from GLRLM (Supplementary Table S3), 5 were from GLSZM (Supplementary Table S4), and 7 were from GLDM (Supplementary Table S5).

### 3.3. Features Extracted from Filtered MR Images of the Pancreas

The filtration of MR images using Laplacian of Gaussian filter with 2mm σ resulted in a total of 33 features that showed a statistically significant difference between the groups (Table 2). These included 5 first-order (Supplementary Table S1) and 28 second-order texture features. Out of the latter, 11 were from GLCM (Supplementary Table S2), 6 were from GLRLM (Supplementary Table S3), 5 were from GLSZM (Supplementary Table S4), 5 were from GLDM (Supplementary Table S5), and 1 was from NGTDM (Supplementary Table S6). When 5mm σ was used, a total of 5 features showed a statistically significant difference between the groups (Table 2). These included 3 first-order (Supplementary Table S1) and 2 second-order texture features: 1 from GLDM (Supplementary Table S5) and 1 from NGTDM (Supplementary Table S6).

The filtration of MR images using logarithm filter resulted in a total of 39 features that showed a statistically significant difference between the groups (Table 2). These included 7 first-order (Supplementary Table S1) and 32 second-order texture features. Out of the latter, 12 were from GLCM (Supplementary Table S2), 8 were from GLRLM (Supplementary Table S3), 7 were from GLSZM (Supplementary Table S4), and 5 were from GLDM (Supplementary Table S5).

## 4. Discussion

The present research was the first to study the effect of 7 different intensity discretization and image filtration configurations on 3T MR images of the pancreas. The investigated pre-processing configurations influenced differentially the number of significant texture features of the pancreas that distinguished individuals with healthy pancreas and those with definite chronic pancreatitis, who were matched on age and sex. The matching was a distinct strength of the present study, not carried out in previous radiomics of the pancreas studies, as morphology of the pancreas varies greatly with age and sex [32,33,34]. In addition, other characteristics of participants in the groups did not differ significantly (Table 1). We documented that the type and parameters of intensity discretization and filtration have a differing effect on the first-order and second-order texture features of the pancreas.

Radiomics of the pancreas is a potential “game-changer” in the management of diffuse diseases of the pancreas. However, it is not ready yet to be translated into routine clinical practice due to the lack of standardization of the specific steps that make up the radiomics pipeline. These include image acquisition protocols, definitions of quantitative radiomics features, and approaches to image pre-processing [35,36,37,38]. Several efforts have recently been made to optimize the use of radiomics. For example, the Quantitative Imaging Biomarker Alliance proposed a set of standardized image acquisition protocols [39]. Further, 169 radiomics features with reference values have been standardized based on the definitions developed by the Image Biomarker Standardisation Initiative [40]. Notably, the above-mentioned projects did not attempt to determine modality-specific, let alone organ-specific, image pre-processing configurations. The present study has made the first step towards addressing this gap in knowledge in regard to pancreas MR images.

As far as intensity discretization is concerned, it can be carried out by means of either absolute discretization (i.e., using fixed bin size) or relative discretization (i.e., using fixed bin number). Our findings support the latter in the context of pancreas MR images. This is because, while the number of significant first-order texture features was not affected by absolute versus relative discretization, the number of second-order texture features differed markedly. Interestingly, similar inference was drawn from the findings of a radiomics of computed tomography images in individuals with locally advanced rectal cancer [41]. In the present study, relative discretization (using fixed bin number of 16) yielded a total of 42 significant second-order features whereas absolute discretization at most yielded only 26 significant second-order features (using fixed bin width of 42). In considering relative discretization, fixed bin number of 16 appeared slightly more advantageous in comparison with fixed bin number of 128 as it increased the number of significant GLCM and GLRLM features (though not the other second-order or first-order texture features). The superiority of relative over absolute discretization demonstrated in the present study is in line with the Image Biomarker Standardisation Initiative guidelines that also recommend the use of relative discretization when dealing with qualitative gray level images [40]. As far as filtering is concerned, Laplacian of Gaussian filter with 5 mm σ clearly underperformed in comparison with the other studied filters/configurations (perhaps, because the pancreas texture heterogeneity is less coarse than 5 mm and is hence not accentuated with this filter). Therefore, its application to pancreas MR images is discouraged. Logarithm filter appeared slightly more advantageous in comparison with Laplacian of Gaussian filter with 2 mm σ as it increased the number of significant first-order texture features and also GLCM, GLRLM, and GLSZM features (though not GLDM or NGTDM features).

While reporting on total number of significant features enabled a high-level triage of dozens texture features that are extractable but not necessarily usable, it is also important to consider differences in specific significant radiomics of the pancreas features. Out of the studied first-order texture features (Supplementary Table S1), the most notable finding related to kurtosis. It was significantly different between the two study groups in six out of the seven pre-processing configurations, with the only non-significant difference being observed when Laplacian of Gaussian filter with 5 mm σ was applied (as discussed above, the use of this configuration in pre-processing of pancreas MR images is not helpful). When the other six configurations were used, kurtosis was consistently lower in individuals with chronic pancreatitis. Low kurtosis signified a more uniform texture of the pancreas in the present study, most likely due to widespread fibrosis of the organ. Out of the studied second-order texture features, we noted significant changes in GLDM features between the two groups (Supplementary Table S5). In particular, gray level non uniformity was significantly higher in individuals with healthy pancreas when 5 (all filtering and absolute discretization) out of the 7 pre-processing configurations were applied. This parameter reflects the difference of gray levels in the image where a higher value correlates with a greater difference in image intensity values [40]. Higher values of this parameter in healthy individuals than those with chronic pancreatitis may reflect a heterogeneous and complex composition of the healthy pancreas that is composed of endocrine, acinar, and ductal cells, as well as intra-pancreatic fat [34,42,43].

The study had several limitations. The effect of only 7 specific configurations of intensity discretization and filtration were investigated. It is acknowledged that the choice of intensity levels (6 and 42), bin numbers (16 and 128), and σ values (2 and 5 mm) was arbitrary. Other types of image filters and other values of bin widths and numbers may need to be investigated in the future radiomics studies of the pancreas [44,45]. As research on radiomics of the pancreas is in its infancy, it is unknown whether the selected configurations were optimal. However, the texture features extracted using the chosen configurations are commonly used and have also been reported to be associated with reproducible radiomics features [29,46]. Further, there are other approaches to relative intensity discretization such as z-score normalization [47]. Although a total of 7 configurations were investigated in the present study, only 1 configuration at a time was studied and reported. Specifically, a limitation of the present study is that, when assessing the effect of image filtering, we did not perform image discretization. A large number of texture features was tested, which might have led to the multiple comparisons issue. However, all the presented analyses took into account false discovery rate. The study exclusively used T1-weighted out-of-phase MR images. Investigation of other images is warranted as the type of image may affect radiomics features [35,48,49]. A VIBE sequence, characterized by lower signal characteristics of fluid compared with standard T1-weighted images, was used in the present study. However, individuals with fluid collections in the pancreas were excluded. The use of radiomic features from T2 maps was shown to be less sensitive to image preprocessing than those from T1 maps and this needs to be explored in future studies on radiomics of the pancreas [50,51]. In addition, it is acknowledged that our findings may not hold true at 1.5 T as MR image contrast can vary with static magnetic field strength. Last, the study sample size (n = 30) was limited. However, end-stage chronic pancreatitis is uncommon with the worldwide incidence of merely 9 cases per 100,000 population [3]. Further, the present study employed the strict eligibility criteria, in particular excluding those individuals with chronic pancreatitis who had pancreatic cysts (in order to minimize the influence of heterogeneous lesions on radiomics of the pancreas). All consecutive participants were enrolled, minimizing the risk of selection bias. It is also worth noting that some published radiomics of the pancreas studies had a smaller sample size [52,53].

In conclusion, filtering of pancreas MR images (in particular, with the use of logarithm filter) has the most pronounced effect on first-order texture features whereas intensity discretization (in particular, with the use of fixed bin number of 16)—on second-order texture features. This information will facilitate the development of a clinically useful radiomics signature of the pancreas in the future.

## Figures and Tables

**Table 1:** Characteristics of the study groups.

Characteristic	Chronic Pancreatitis (*n* = 15)	Health (*n* = 15)	*p*
Men, *n* (%)	12 (80.0)	12 (80.0)	1.000
Age (years)	59.9 ± 10.9	59.8 ± 11.3	0.987
Body mass index (kg/m^2^)	28.7 ± 6.2	24.9 ± 4.4	0.070
Weight (kg)	83.8 ± 17.2	76.7 ± 16.1	0.253
Height (cm)	171.6 ± 10.7	175.1 ± 10.3	0.368
HDL cholesterol (mmol/L)	1.5 ± 0.4	1.4 ± 0.5	0.785
LDL cholesterol (mmol/L)	2.4 ± 1.1	2.8 ± 0.6	0.225
Total cholesterol (mmol/L)	4.8 ± 1.3	4.6 ± 0.9	0.705
HOMA-IR (mIU/L·mmol/L)	111.8 ± 150.5	36.2 ± 31.5	0.067
Fasting insulin (mIU/L)	20.4 ± 27.5	13.8 ± 10.9	0.452

*Footnote*: Data are presented as mean ± standard error or percentage. *Abbreviations*: HDL, high density lipoprotein; LDL, low density lipoprotein; HOMA-IR, homeostatic model assessment of insulin resistance.

**Table 2:** Number of statistically significantly different texture features between the two study groups with the use of the studied pre-processing configurations.

Feature	Intensity Discretization				Filtration		
	FBN 16	FBN 128	FBW 6	FBW 42	2 mm σ	5 mm σ	Logarithm
First-order texture	3	3	3	3	5	3	7
GLCM	15	12	4	6	11	0	12
GLRLM	11	8	6	8	6	0	8
GLSZM	6	9	6	5	5	0	7
GLDM	9	8	8	7	5	1	5
NGTDM	1	1	0	0	1	1	0

*Footnotes*: 2 mm σ and 5 mm σ refer to the configurations of Laplacian of Gaussian filter. Statistically significance was determined after accounting for false discovery rate. *Abbreviations*: FBN, fixed bin number; FBW, fixed bin width; GLCM, gray level co-occurrence matrix; GLDM, gray level dependence matrix; GLRLM, gray level run length matrix; GLSZM, gray level size zone matrix; NGTDM, neighboring gray tone difference matrix.

## Data Availability

The data supporting the findings of this study are available within the article and its Supplementary Materials.

## Supplementary Materials

The following supporting information can be downloaded at: https://www.mdpi.com/article/10.3390/jimaging8080220/s1, Table S1: First-order texture features that showed a statistically significant difference between the study groups; Table S2: GLCM texture features that showed a statistically significant difference between the study groups; Table S3: GLRLM texture features that showed a statistically significant difference between the study groups; Table S4: GLSZM texture features that showed a statistically significant difference between the study groups; Table S5: GLDM texture features that showed a statistically significant difference between the study groups; Table S6: NGTDM texture features that showed a statistically significant difference between the study groups.
