# Supplementary material for: Effect of Gray Value Discretization and Image Filtration on Texture Features of the Pancreas Derived from Magnetic Resonance Imaging at 3T
Source: J Imaging. 2022 Aug 18;8(8):220. doi: 10.3390/jimaging8080220 (PMC9409719; doi:10.3390/jimaging8080220)
Supplement: Supplementary file 1 [file jimaging-08-00220-s001.zip › jimaging-1813749-supplementary.pdf]

Supplementary Materials

Supplementary Table S1. First-order texture features that showed a statistically significant difference between the study groups

Image type	Feature name	Chronic pancreatitis	Health	p	q
Intensity discretized (FBN 16)	Kurtosis	2.87 ± 0.46	3.26 ± 0.54	0.039	0.044
	Energy (10^8)	9.09 ± 6.09	14.27 ± 7.25	0.043	0.044
	10th percentile	68.51 ± 20.69	111.98 ± 46.92	0.004	0.015
Intensity discretized (FBN 128)	Kurtosis	2.87 ± 0.46	3.26 ± 0.54	0.039	0.044
	Energy (10^8)	9.09 ± 6.09	14.27 ± 7.25	0.043	0.044
	10th percentile	68.51 ± 20.69	111.98 ± 46.92	0.004	0.015
Intensity discretized (FBW 6)	Kurtosis	2.87 ± 0.46	3.26 ± 0.54	0.039	0.044
	Energy (10^8)	9.09 ± 6.09	14.27 ± 7.25	0.043	0.044
	10th percentile	68.51 ± 20.69	111.98 ± 46.92	0.004	0.015
Intensity discretized (FBW 42)	Kurtosis	2.87 ± 0.46	3.26 ± 0.54	0.039	0.044
	Energy (10^8)	9.09 ± 6.09	14.27 ± 7.25	0.043	0.044
	10th percentile	68.51 ± 20.69	111.98 ± 46.92	0.004	0.015
2 mm σ LoG filter	Mean	1.26 ± 5.92	−4.93 ± 7.73	0.020	0.035
	Kurtosis	3.11 ± 0.39	3.78 ± 0.91	0.017	0.035
	90th percentile	58.74 ± 12.47	43.56 ± 17.98	0.013	0.032
	Interquartile range	58.61 ± 12.82	47.63 ± 15.29	0.042	0.044
	Robust mean absolute deviation	24.37 ± 5.24	19.99 ± 6.26	0.048	0.048
5 mm σ LoG filter	Mean	0.27 ± 11.71	−15.05 ± 16.57	0.007	0.021
	Median	1.84 ± 11.74	−15.37 ± 17.68	0.004	0.015
	90th percentile	37.79 ± 9.74	22.93 ± 13.61	0.002	0.015
Logarithm filter	Entropy	7.79 ± 0.28	7.42 ± 0.37	0.005	0.017
	Uniformity	0.0056 ± 0.0013	0.0078 ± 0.0023	0.004	0.015
	Skewness	−2.92 ± 0.41	−3.53 ± 0.88	0.024	0.038
	Kurtosis	16.61 ± 3.61	26.67 ± 12.72	0.009	0.024
	Interquartile range	97.22 ± 46.63	62.82 ± 26.63	0.019	0.035
	Mean absolute deviation	73.59 ± 35.34	48.53 ± 18.21	0.021	0.035
	Robust mean absolute deviation	42.57 ± 20.38	27.42 ± 11.44	0.018	0.035

Footnotes: Data are presented as mean ± standard error. q value represents the minimum false discovery rate at which a result can be considered significant.
Abbreviations: FBN, fixed bin number; FBW, fixed bin width.

Supplementary Table S2. GLCM texture features that showed a statistically significant difference between the study groups

Image type	Feature name	Chronic pancreatitis	Health	p	q
Intensity discretized (FBN 16)	Contrast	8.24 ± 2.09	6.43 ± 2.44	0.038	0.046
	Correlation	0.39 ± 0.06	0.48 ± 0.09	0.008	0.037
	Autocorrelation	46.05 ± 15.64	62.23 ± 21.05	0.024	0.045
	Sum average	12.98 ± 2.33	15.17 ± 2.85	0.029	0.045
	Joint average	6.49 ± 1.17	7.59 ± 1.43	0.029	0.045
	Inverse variance	0.37 ± 0.03	0.39 ± 0.04	0.037	0.046
	Inverse difference	0.47 ± 0.04	0.52 ± 0.07	0.028	0.045
	Difference average	2.09 ± 0.32	1.78 ± 0.43	0.029	0.045
	Difference entropy	2.59 ± 0.18	2.42 ± 0.28	0.045	0.047
	Inverse difference moment	0.39 ± 0.05	0.46 ± 0.08	0.028	0.045
	Maximal correlation coefficient	0.43 ± 0.08	0.51 ± 0.11	0.025	0.045
	Inverse difference normalized	0.89 ± 0.01	0.91 ± 0.02	0.028	0.045
	Inverse difference moment normalized	0.97 ± 0.01	0.98 ± 0.01	0.035	0.046
	Information measure of correlation 1	−0.08 ± 0.02	−0.11 ± 0.05	0.040	0.047
	Information measure of correlation 2	0.49 ± 0.09	0.59 ± 0.12	0.018	0.045
Intensity discretized (FBN 128)	Contrast	520.32 ± 134.78	403.52 ± 158.98	0.039	0.046
	Correlation	0.39 ± 0.07	0.48 ± 0.10	0.008	0.037
	Joint average	48.38 ± 9.29	57.15 ± 11.42	0.029	0.045
	Sum average	96.77 ± 18.59	114.29 ± 22.84	0.029	0.045
	Inverse variance	0.07 ± 0.02	0.09 ± 0.03	0.028	0.045
	Difference average	16.84 ± 2.53	14.29 ± 3.49	0.030	0.045
	Difference entropy	5.41 ± 0.21	5.17 ± 0.37	0.042	0.047
	Autocorrelation (10^2)	25.93 ± 9.37	35.69 ± 12.69	0.024	0.045
	Inverse difference	0.14 ± 0.02	0.17 ± 0.04	0.029	0.045
	Inverse difference moment	0.07 ± 0.02	0.09 ± 0.03	0.029	0.045
	Inverse difference normalized	0.89 ± 0.01	0.91 ± 0.02	0.029	0.045
	Inverse difference moment normalized	0.97 ± 0.01	0.98 ± 0.01	0.036	0.046
Intensity discretized (FBW 6)	Correlation	0.39 ± 0.07	0.48 ± 0.10	0.008	0.037
	Maximal correlation coefficient	0.45 ± 0.08	0.52 ± 0.11	0.047	0.047
	Inverse difference normalized	0.89 ± 0.01	0.91 ± 0.02	0.029	0.045
	Inverse difference moment normalized	0.97 ± 0.01	0.98 ± 0.01	0.037	0.046
Intensity discretized (FBW 42)	Correlation	0.39 ± 0.07	0.48 ± 0.10	0.007	0.037
	Maximal correlation coefficient	0.42 ± 0.08	0.49 ± 0.11	0.023	0.045
	Inverse difference normalized	0.89 ± 0.01	0.91 ± 0.02	0.030	0.045

	Inverse difference moment normalized	0.97 ± 0.01	0.98 ± 0.01	0.038	0.046
	Information measure of correlation 1	−0.09 ± 0.02	−0.12 ± 0.05	0.045	0.048
	Information measure of correlation 2	0.49 ± 0.09	0.58 ± 0.11	0.014	0.045
2 mm σ LoG filter	Contrast (10^2)	28.76 ± 12.27	19.89 ± 10.76	0.044	0.048
	Correlation	0.25 ± 0.07	0.33 ± 0.07	0.002	0.037
	Inverse variance	0.05 ± 0.02	0.06 ± 0.02	0.046	0.048
	Inverse difference	0.09 ± 0.02	0.11 ± 0.03	0.046	0.048
	Difference average	37.49 ± 8.56	30.33 ± 9.01	0.034	0.046
	Difference entropy	6.43 ± 0.32	6.14 ± 0.44	0.046	0.048
	Inverse difference moment	0.05 ± 0.02	0.06 ± 0.02	0.048	0.048
	Inverse difference normalized	0.90 ± 0.01	0.92 ± 0.02	0.012	0.045
	Inverse difference moment normalized	0.97 ± 0.01	0.99 ± 0.01	0.005	0.037
	Information measure of correlation 1	−0.19 ± 0.05	−0.15 ± 0.04	0.020	0.045
	Information measure of correlation 2	0.96 ± 0.03	0.92 ± 0.05	0.006	0.037
Logarithm filter	Correlation	0.31 ± 0.05	0.39 ± 0.11	0.015	0.045
	Joint energy	0.000098 ± 0.000044	0.00015 ± 0.000077	0.034	0.046
	Sum entropy	8.84 ± 0.54	8.31 ± 0.53	0.012	0.045
	Inverse variance	0.03 ± 0.02	0.05 ± 0.03	0.004	0.037
	Inverse difference	0.067 ± 0.018	0.099 ± 0.035	0.004	0.037
	Difference average	72.07 ± 32.75	44.87 ± 23.98	0.015	0.045
	Difference entropy	7.35 ± 0.55	6.59 ± 0.78	0.004	0.037
	Maximum probability	0.00058 ± 0.00019	0.00081 ± 0.00033	0.039	0.046
	Inverse difference moment	0.028 ± 0.009	0.048 ± 0.022	0.004	0.037
	Inverse difference normalized	0.93 ± 0.02	0.95 ± 0.03	0.025	0.045
	Information measure of correlation 1	−0.22 ± 0.055	−0.17 ± 0.034	0.006	0.037
	Information measure of correlation 2	0.98 ± 0.02	0.95 ± 0.03	0.001	0.037

Footnotes: Data are presented as mean ± standard error. q value represents the minimum false discovery rate at which a result can be considered significant.
Abbreviations: FBN, fixed bin number; FBW, fixed bin width; GLCM, gray level co-occurrence matrix.

Supplementary Table S3. GLRLM texture features that showed a statistically significant difference between the study groups

Image type	Feature name	Chronic pancreatitis	Health	p	q
Intensity discretized (FBN 16)	Run entropy	4.09 ± 0.11	4.21 ± 0.14	0.017	0.043
	Run variance	0.28 ± 0.11	0.48 ± 0.33	0.033	0.043
	Run percentage	0.86 ± 0.04	0.81 ± 0.06	0.022	0.043
	Long run emphasis	1.71 ± 0.25	2.12 ± 0.63	0.030	0.043
	Short run emphasis	0.89 ± 0.03	0.85 ± 0.04	0.022	0.043
	High gray level run emphasis	47.38 ± 14.22	61.76 ± 18.27	0.023	0.043
	Low gray level run emphasis	0.09 ± 0.03	0.06 ± 0.04	0.032	0.043
	Short run low gray level emphasis	0.09 ± 0.03	0.06 ± 0.04	0.027	0.043
	Short run high gray level emphasis	41.89 ± 12.51	52.22 ± 14.39	0.045	0.047
	Long run high gray level emphasis	81.51 ± 26.09	136.19 ± 61.61	0.005	0.026
	Run length non uniformity normalized	0.76 ± 0.05	0.71 ± 0.07	0.017	0.043
Intensity discretized (FBN 128)	Run variance	0.02 ± 0.01	0.03 ± 0.01	0.026	0.043
	Run percentage	0.98 ± 0.01	0.97 ± 0.01	0.024	0.043
	Short run emphasis	0.99 ± 0.01	0.98 ± 0.01	0.023	0.043
	Long run emphasis	1.06 ± 0.02	1.08 ± 0.03	0.025	0.043
	High gray level run emphasis (10^2)	27.06 ± 8.83	35.85 ± 11.41	0.026	0.043
	Run length non uniformity normalized	0.97 ± 0.01	0.95 ± 0.02	0.023	0.043
	Short run high gray level emphasis (10^2)	26.65 ± 8.69	35.09 ± 11.08	0.028	0.043
	Long run high gray level emphasis (10^2)	28.78 ± 9.44	39.13 ± 12.92	0.018	0.043
Intensity discretized (FBW 6)	Run variance	0.04 ± 0.01	0.05 ± 0.03	0.036	0.043
	Run percentage	0.98 ± 0.01	0.96 ± 0.02	0.033	0.043
	Long run emphasis	1.09 ± 0.03	1.14 ± 0.07	0.036	0.043
	Short run emphasis	0.98 ± 0.01	0.97 ± 0.01	0.034	0.043
	Gray level non uniformity (10^2)	3.85 ± 1.62	5.47 ± 1.86	0.017	0.043
	Run length non uniformity normalized	0.95 ± 0.01	0.93 ± 0.03	0.032	0.043
Intensity discretized (FBW 42)	Run entropy	3.87 ± 0.16	4.01 ± 0.19	0.027	0.043
	Run variance	0.45 ± 0.21	0.84 ± 0.69	0.048	0.048
	Run percentage	0.81 ± 0.04	0.76 ± 0.08	0.032	0.043
	Long run emphasis	2.08 ± 0.43	2.83 ± 1.27	0.047	0.048
	Short run emphasis	0.86 ± 0.03	0.83 ± 0.06	0.036	0.043
	Short run low gray level emphasis	0.12 ± 0.04	0.08 ± 0.05	0.039	0.043
	Long run high gray level emphasis	60.14 ± 20.19	107.98 ± 52.67	0.004	0.026
	Run length non uniformity normalized	0.70 ± 0.05	0.65 ± 0.08	0.029	0.043
2 mm σ LoG filter	Run variance	0.01 ± 0.01	0.02 ± 0.01	0.041	0.043
	Run percentage	0.99 ± 0.01	0.98 ± 0.01	0.038	0.043
	Short run emphasis	0.99 ± 0.01	0.98 ± 0.01	0.039	0.043
	Long run emphasis	1.04 ± 0.02	1.05 ± 0.03	0.040	0.044
	Gray level non uniformity	128.07 ± 53.79	211.42 ± 98.13	0.007	0.030

	Run length non uniformity normalized	0.98 ± 0.01	0.97 ± 0.02	0.038	0.043
Logarithm filter	Run entropy	7.84 ± 0.27	7.52 ± 0.34	0.006	0.028
	Run variance	0.01 ± 0.01	0.02 ± 0.01	0.005	0.026
	Run percentage	0.99 ± 0.01	0.98 ± 0.01	0.004	0.026
	Short run emphasis	0.99 ± 0.01	0.98 ± 0.01	0.004	0.026
	Long run emphasis	1.02 ± 0.01	1.05 ± 0.03	0.005	0.026
	Gray level non uniformity	103.59 ± 42.44	189.86 ± 79.64	0.001	0.026
	Gray level non uniformity normalized	0.0056 ± 0.0013	0.0077 ± 0.0022	0.004	0.026
	Run length non uniformity normalized	0.99 ± 0.01	0.97 ± 0.02	0.004	0.026

Footnotes: Data are presented as mean ± standard error. q value represents the minimum false discovery rate at which a result can be considered significant.

Abbreviations: FBN, fixed bin number; FBW, fixed bin width; GLRLM, gray level run length matrix.

Supplementary Table S4. GLSZM texture features that showed a statistically significant difference between the study groups

Image type	Feature name	Chronic pancreatitis	Health	p	q
Intensity discretized (FBN 16)	Zone percentage	0.15 ± 0.03	0.11 ± 0.04	0.014	0.031
	High gray level zone emphasis	46.09 ± 6.85	52.94 ± 7.45	0.014	0.031
	Low gray level zone emphasis	0.12 ± 0.03	0.08 ± 0.03	0.005	0.023
	Small area high gray level emphasis	24.72 ± 3.61	27.91 ± 4.01	0.030	0.040
	Small area low gray level emphasis	0.06 ± 0.01	0.05 ± 0.01	0.003	0.021
	Large area high gray level emphasis (10^5)	2.43 ± 4.02	8.32 ± 8.29	0.022	0.033
Intensity discretized (FBN 128)	Zone entropy	7.33 ± 0.09	7.48 ± 0.16	0.004	0.021
	Zone percentage	0.78 ± 0.05	0.72 ± 0.08	0.020	0.033
	Small area emphasis	0.85 ± 0.03	0.81 ± 0.04	0.016	0.033
	Low gray level zone emphasis	0.010 ± 0.004	0.007 ± 0.005	0.046	0.047
	Small area low gray level emphasis	0.008 ± 0.003	0.005 ± 0.004	0.029	0.040
	Size zone non uniformity normalized	0.67 ± 0.04	0.63 ± 0.06	0.014	0.031
	High gray level zone emphasis (10^2)	26.79 ± 8.29	35.32 ± 10.71	0.021	0.033
	Small area high gray level emphasis (10^2)	22.54 ± 6.86	28.78 ± 7.91	0.043	0.046
	Large area high gray level emphasis (10^3)	6.33 ± 2.24	13.99 ± 9.79	0.010	0.031
Intensity discretized (FBW 6)	Zone entropy	7.06 ± 0.15	7.25 ± 0.19	0.006	0.025
	Zone percentage	0.68 ± 0.07	0.61 ± 0.11	0.030	0.040
	Small area emphasis	0.79 ± 0.04	0.76 ± 0.05	0.041	0.046
	Small area low gray level emphasis	0.02 ± 0.004	0.007 ± 0.006	0.034	0.043
	Size zone non uniformity normalized	0.58 ± 0.05	0.54 ± 0.07	0.043	0.046
	Large area high gray level emphasis (10^3)	4.66 ± 2.05	19.81 ± 24.67	0.033	0.043
Intensity discretized (FBW 42)	Zone variance (10^4)	1.43 ± 1.25	4.12 ± 3.87	0.020	0.033
	Large area emphasis (10^3)	14.44 ± 12.54	41.44 ± 38.93	0.020	0.033
	Low gray level zone emphasis	0.17 ± 0.04	0.14 ± 0.04	0.018	0.033
	Small area low gray level emphasis	0.08 ± 0.02	0.06 ± 0.02	0.011	0.031
	Large area high gray level emphasis (10^5)	4.04 ± 3.97	14.26 ± 9.23	0.012	0.031
2 mm σ LoG filter	Zone percentage	0.87 ± 0.04	0.83 ± 0.07	0.037	0.045
	Large area emphasis	1.59 ± 0.24	1.97 ± 0.64	0.048	0.048
	Small area emphasis	0.91 ± 0.03	0.89 ± 0.04	0.044	0.046
	Gray level non uniformity	109.09 ± 46.32	162.69 ± 58.67	0.010	0.031
	Size zone non uniformity normalized	0.79 ± 0.05	0.74 ± 0.07	0.044	0.046
	Zone variance	0.17 ± 0.07	0.42 ± 0.36	0.017	0.033

	Zone percentage	0.89 ± 0.026	0.84 ± 0.067	0.004	0.021
	Small area emphasis	0.93 ± 0.02	0.89 ± 0.04	0.003	0.021
	Large area emphasis	1.41 ± 0.14	1.86 ± 0.59	0.011	0.031
Logarithm filter	Gray level non uniformity	89.74 ± 35.62	146.02 ± 50.05	0.001	0.021
	Size zone non uniformity normalized	0.84 ± 0.034	0.76 ± 0.076	0.003	0.021
	Gray level non uniformity normalized	0.0054 ± 0.0010	0.0071 ± 0.0019	0.004	0.021

Footnotes: Data are presented as mean ± standard error. q value represents the minimum false discovery rate at which a result can be considered significant.

Abbreviations: FBN, fixed bin number; FBW, fixed bin width; GLSZM, gray level size zone matrix.

Supplementary Table S5. GLDM texture features that showed a statistically significant difference between the study groups

Image type	Feature name	Chronic pancreatitis	Health	p	q
Intensity discretized (FBN 16)	Dependence entropy	6.59 ± 0.14	6.79 ± 0.23	0.009	0.027
	Dependence variance	7.92 ± 3.65	12.79 ± 6.84	0.024	0.042
	High gray level emphasis	47.74 ± 14.81	62.41 ± 19.06	0.026	0.042
	Low gray level emphasis	0.09 ± 0.04	0.06 ± 0.05	0.038	0.042
	Small dependence emphasis	0.15 ± 0.03	0.12 ± 0.04	0.018	0.035
	Large dependence emphasis	32.59 ± 14.31	50.03 ± 25.03	0.028	0.042
	Dependence non uniformity normalized	0.12 ± 0.01	0.09 ± 0.03	0.011	0.031
	Small dependence low gray level emphasis	0.02 ± 0.003	0.01 ± 0.005	0.005	0.019
	Large dependence high gray level emphasis (10^2)	15.83 ± 6.68	34.13 ± 20.22	0.004	0.019
Intensity discretized (FBN 128)	Dependence entropy	7.67 ± 0.11	7.82 ± 0.17	0.009	0.027
	Dependence variance	0.61 ± 0.19	0.88 ± 0.43	0.030	0.042
	Small dependence emphasis	0.71 ± 0.05	0.64 ± 0.08	0.017	0.034
	Large dependence emphasis	2.92 ± 0.62	3.79 ± 1.29	0.029	0.042
	High gray level emphasis (10^2)	27.07 ± 8.87	35.87 ± 11.45	0.026	0.042
	Dependence non uniformity normalized	0.48 ± 0.05	0.41 ± 0.08	0.014	0.032
	Small dependence low gray level emphasis	0.007 ± 0.003	0.005 ± 0.004	0.043	0.044
	Large dependence high gray level emphasis (10^3)	7.95 ± 2.93	14.31 ± 7.13	0.005	0.019
Intensity discretized (FBW 6)	Dependence entropy	7.41 ± 0.15	7.59 ± 0.19	0.013	0.032
	Dependence variance	0.96 ± 0.31	1.49 ± 0.88	0.037	0.042
	Small dependence emphasis	0.60 ± 0.06	0.54 ± 0.09	0.032	0.042
	Large dependence emphasis	4.13 ± 0.99	5.82 ± 2.73	0.037	0.042
	Gray level non uniformity (10^2)	3.99 ± 1.68	5.79± 2.05	0.014	0.031
	Dependence non uniformity normalized	0.38 ± 0.05	0.32 ± 0.08	0.030	0.042
	Small dependence low gray level emphasis	0.009 ± 0.003	0.006 ± 0.004	0.042	0.044
	Large dependence high gray level emphasis (10^3)	5.06 ± 1.95	9.67 ± 4.94	0.003	0.019
Intensity discretized (FBW 42)	Dependence entropy	6.46 ± 0.16	6.64 ± 0.21	0.014	0.031
	Dependence variance	11.39 ± 4.57	17.89 ± 9.85	0.031	0.042
	Large dependence emphasis	48.25 ± 17.59	73.93 ± 39.56	0.033	0.042
	Gray level non uniformity (10^3)	2.76 ± 1.16	3.97 ± 1.39	0.015	0.032
	Dependence non uniformity normalized	0.09 ± 0.02	0.08 ± 0.03	0.024	0.042
	Small dependence low gray level emphasis	0.014 ± 0.003	0.009 ± 0.005	0.001	0.019
	Large dependence high gray level emphasis (10^2)	14.12 ± 5.74	29.37 ± 16.18	0.003	0.019
2 mm σ LoG filter	Dependence variance	0.33 ± 0.11	0.49 ± 0.25	0.036	0.042
	Gray level non uniformity	129.82 ± 54.48	216.32 ± 102.67	0.007	0.025

	Large dependence emphasis	2.01 ± 0.32	2.45 ± 0.71	0.039	0.042
	Small dependence emphasis	0.82 ± 0.05	0.77 ± 0.08	0.039	0.042
	Dependence non uniformity normalized	0.63 ± 0.07	0.56 ± 0.09	0.038	0.042
5 mm σ LoG filter	Gray level non uniformity	191.94 ± 87.54	261.16 ± 96.38	0.049	0.049
Logarithm filter	Dependence variance	0.24 ± 0.08	0.43 ± 0.23	0.005	0.019
	Gray level non uniformity	104.87 ± 43.12	194.23 ± 82.86	0.001	0.019
	Large dependence emphasis	1.72 ± 0.22	2.31 ± 0.68	0.005	0.019
	Small dependence emphasis	0.86 ± 0.04	0.79 ± 0.08	0.003	0.019
	Dependence non uniformity normalized	0.69 ± 0.06	0.59 ± 0.12	0.003	0.019

Footnotes: Data are presented as mean ± standard error. q value represents the minimum false discovery rate at which a result can be considered significant.
Abbreviations: FBN, fixed bin number; FBW, fixed bin width; GLDM, gray level dependence matrix.

Supplementary Table S6. NGTDM texture features that showed a statistically significant difference between the study groups

Image type	Feature name	Chronic pancreatitis	Health	p	q
Intensity discretized (FBN 16)	Complexity	168.94 ± 21.13	138.45 ± 39.43	0.015	0.032
Intensity discretized (FBN 128)	Contrast	0.75 ± 0.23	0.56 ± 0.23	0.032	0.032
2 mm σ LoG filter	Contrast	1.24 ± 0.48	0.84 ± 0.42	0.021	0.032
5 mm σ LoG filter	Coarseness	0.00075 ± 0.00034	0.00051 ± 0.00015	0.025	0.032

Footnotes: Data are presented as mean ± standard error. q value represents the minimum false discovery rate at which a result can be considered significant.

Abbreviations: FBN, fixed bin number; NGTDM, neighbouring gray tone difference matrix.
